# Multigram-Scale Synthesis of Luminescent Cesium Lead Halide Perovskite Nanobricks for Plastic Scintillators

**DOI:** 10.1021/acsanm.3c01146

**Published:** 2023-05-31

**Authors:** Sara Mecca, Francesca Pallini, Valerio Pinchetti, Andrea Erroi, Alice Fappani, Francesca Rossi, Sara Mattiello, Giovanni Maria Vanacore, Sergio Brovelli, Luca Beverina

**Affiliations:** † Department of Materials Science, University of Milano-Bicocca, via R. Cozzi 55, I-20126 Milan, Italy; ‡ IMEM-CNR Institute, Parco Area delle Scienze 37/A, 43124 Parma, Italy

**Keywords:** Luminescent nanocrystals, perovskites, scaling up, colloidal synthesis, recycling

## Abstract

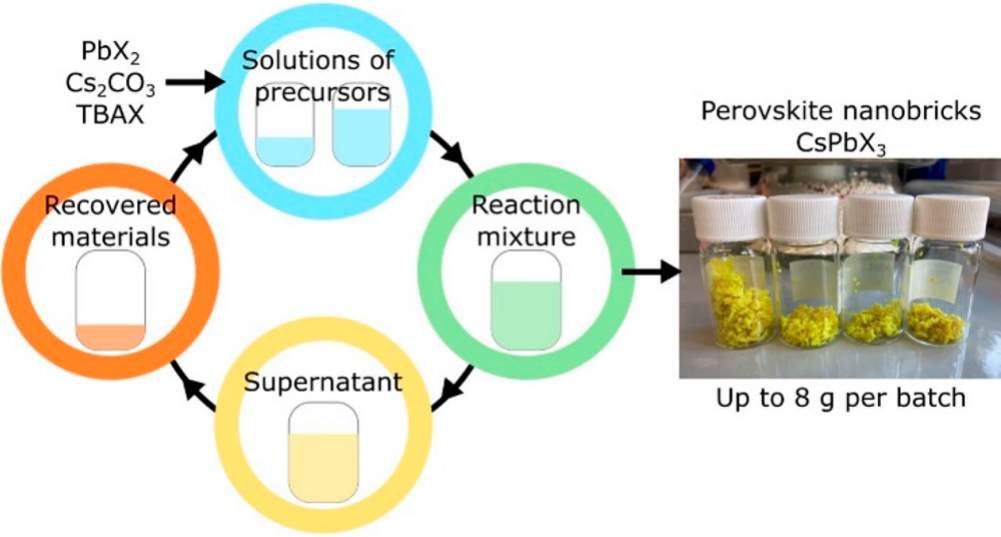

Cesium lead halide perovskite nanocrystals of general formula CsPbX_3_ are having tremendous impact on a vast array of technologies requiring strong and tunable luminescence across the visible range and solutions processing. The development of plastic scintillators is just one of the many relevant applications. The syntheses are relatively simple but generally unsuitable to produce a large amount of material of reproducible quality required when moving from proof-of-concept scale to industrial applications. Wastes, particularly large amounts of lead-contaminated toxic and flammable organic solvents, are also an open issue. We describe a simple and reproducible procedure enabling the synthesis of luminescent CsPbX_3_ nanobricks of constant quality on a scale going from 0.12 to 8 g in a single batch. We also show complete recycling of the reaction wastes, leading to dramatically improved efficiency and sustainability.

## Introduction

Inorganic lead halide perovskite nanocrystals (LHP-NCs) of general formula CsPbX_3_, where X is a halogen atom, are generating vast scientific and technological interest as luminescent materials.^1^ Depending on the composition and synthetic route, the luminescence of LHP-NCs can be widely tuned in the visible range and reach emission efficiencies close to 100%.^2−4^ The combination of such relevant performances with solution processability makes these materials particularly suitable for most (opto)electronic device platforms, including photovoltaics,^5,6^ radiation and photodetectors,^7−9^ lasers,^10,11^ light-emitting devices,^12^ and luminescent solar concentrators.^13−15^ We have been increasingly focusing on plastic scintillators, requiring particularly large amounts of material to reach suitable cross sections for ionizing radiations.^9^ The availability of reliable, scalable, and possibly sustainable syntheses of LHP-NCs is critical for industrial success. Since the first report on LHP-NCs in 2015 by Protesescu et al.,^16^ the in-solution, hot-injection method has remained the most popular synthetic procedure leading to nanosized crystals with different morphologies, such as nanocubes,^17^ nanoplatelets,^18,19^ and nanowires.^20^

Albeit successful on a lab scale, the hot injection method is ill defined for scaling up. It is particularly difficult to avoid concentration profiles while performing the injection of precursors using large volumes of solutions. The need for inert atmosphere and high temperatures are additional concerns. To overcome such limitations, in 2016, Li et al.^21^ adapted the ligand-assisted reprecipitation process (LARP), previously developed for the synthesis of MaPbX_3_ perovskites,^22^ to the preparation of CsPbX_3_ NCs. This process is simple and efficient even at room temperature. Unfortunately, the preparation of high quality materials requires the use of aprotic dipolar solvents like N,N-dimethylformamide (DMF) or dimethyl sulfoxide (DMSO) whose acceptance in the chemical industry is dwindling.^23^ Moreover, the process does not scale up flawlessly, again due to concentration profiles.^24,25^ Furthermore, elaborate approaches require the use of microemulsion^26^ and reverse emulsion techniques,^27^ ultrasounds,^28^ or mechanochemistry.^29,30^ The breakthrough result toward the development of a robust, reproducible, and scalable method was reported by Akkerman et al., for the first time showcasing room temperature reaction, tolerance to laboratory atmosphere, and compatibility with solvents of lower toxicity.^31^ The method produces colloidal NCs featuring two relatively small molecular weight ligands: propionic acid (PA) and butylamine (BuAm). The choice of ligands was dictated by the target application in optoelectronic devices requiring charge transport capabilities as well as strong luminescence. In terms of colloidal stability, the formulation is poor as aggregation and precipitation happen rather rapidly. Applications requiring stable colloidal dispersions entail replacement of BuAm with oleylamine (OAm) or octylamine and/or the use of fatty acids like octanoic acid.^32,33^ Additional reported modifications of the Akkerman protocol include the tuning of the Cs^+^/Pb^2+^ precursors ratio enabling the gradual change of the NCs morphology from 3D nanocubes to 2D nanoplatelets^34^ and the use of a mixture of PA and OAm along with the hydrobromides of terminal diamines of different chain lengths to control packing while in thin film form.^35^ None of these methods was ever tested for the production of gram-scale batches of NCs of controlled and reproducible quality.

In this work, we describe a highly reproducible, room temperature, and laboratory atmosphere compatible procedure providing compatible results within experimental error over a 2 order of magnitude change in scale, from 120 mg to 8 g of dry CsPbBr_3_ NCs in a single preparation. Such an amount was only limited by the volume of the largest reactor we could use in our laboratory, and there are no fundamental limits for a further increase in the scale. The CsPbBr_3_ NCs we produced have an ∼1.5 aspect ratio, corresponding to a nanobrick (NB) morphology. The protocol can be extended to different halogens (PbCl_2_ and PbI_2_ as precursors in the place of PbBr_2_) and amine ligands (BuAm, instead of OAm). Yield, structural properties, and optical features of NBs are invariant with respect to the scale of the reaction. The original and key enabling step of the procedure is the use of a rotor-stator turboemulsifier in the place of magnetic and overhead stirring, combined with a careful tuning of a previously reported room temperature rapid injection procedure. Our synthesis gives no byproduct distinct from excess reagents (PbBr_2_ and OAm) and solvents. We thus devised a procedure for the complete recovery and reuse of wastes (lead salts in particular). The materials we made using recycled reagents have the same luminescence efficiency of those made using fresh raw materials. Our approach could dramatically help the level of acceptance of lead-containing perovskites at an industrial level, by making them more sustainable and compatible with a circular economy approach.^36^

## Experimental Section

### Chemicals

Cesium carbonate (Cs_2_CO_3_, 99%) and lead(II) bromide (PbBr_2_, 99.99%) were purchased from Fluorochem. Propionic acid (PA, >99.5%) and tetrabutylammonium bromide (TBAB, >98%) were purchased from Merck. Heptane (Hept, Gpr rectapur, 99.8%) and isopropanol (iPrOH, HiPerSolv chromanorm for HPLC >98%) were purchased from VWR. Oleylamine (OAm, 80%–90%) was purchased from Acros Organic. Isopropanol was dried over CaH_2_ for a week and then distilled before use. All other chemicals were used without any further purification and stored in a dryer. Compositions of solvent mixtures are indicated as volume/volume ratios. A turboemulsifier homogenizer was bought from IKA and is composed of a motor group T25/T50 digital Ultra Turrax + dispersing tool with codes S25N-25G and S50N-G45 M for volumes, respectively, up to 2 L and above 2 L.

### Synthesis of CsPbBr_3_ NCs with Turboemulsifier (3.6 L scale, 8 g)

A solution containing the Cs^+^ precursor (from here on defined as solution A) is prepared by reacting Cs_2_CO_3_ (6 mmol, 1.95 g) with PA (6 mL, 79.8 mmol) and then diluting the resulting solution with 3600 mL of Hept/iPrOH 2:1 vol mixture. A solution containing the Pb^2+^ precursor (from here on defined as solution B) is prepared by dissolving PbBr_2_ (60 mmol, 22 g) and TBAB (60 mmol, 19.3 g) in a mixture of OAm (540 mmol, 177.6 mL), PA (540 mmol, 40.2 mL), and iPrOH (60 mL) at 80 °C. After the complete dissolution of the precursors, the mixture is cooled to room temperature (no precipitation observed over 8 h). Solution A is put under stirring with a turboemulsifier homogenizer (15k RPM) in a 5 L beaker, and then, solution B is swiftly poured. The mixture is further homogenized for 30 s to give a clear yellow solution. Upon dilution with 1.8 L of iPrOH, a fine precipitate forms, that is collected by centrifugation at 4500 rpm for 2 min. The supernatant is recovered and recycled as described below, while the bright yellow solid is dried under reduced pressure until constant weight (8 g) and stored in a glovebox under argon atmosphere. No further purification nor size selection steps are performed.

### Synthesis of CsPbBr_3_ NCs with Magnetic Stirring (1.2 L scale)

The procedure is reported in detail in the Supporting Information, Section S4.

### Synthesis of CsPbBr_3_ NCs with Recycled Excess Reagents and Solvents

We selected 120 mL scale. Solution A is prepared with 69 mL of distilled recovered solvents (Hept/iPrOH 1:1.3 v/v), to which we added 51 mL of Hept to reach the desired ratio of Hept/iPrOH 2:1. The amount of Cs_2_CO_3_ required is 62 mg (0.19 mmol) instead of 65 mg, considering the percentage of Cs^+^ evaluated with ICP-OES (0.04 wt %, Table S7) and 0.1 mL of propionic acid (1.3 mmol). The recovered mixture already contains a partial lead source, TBAB (respectively, Pb 2.25 wt % from ICP-OES (Table S7) and approximated 7 wt % from TGA (Figure S14b)), OAm, and PA. We employed 6.6 mL of recovered mixture (6.27 g) and thus added PbBr_2_ (438 mg, 1.32 mmol), TBAB (205 mg, 0.6 mmol), OAm (1.1 mL, 3.3 mmol), and iPrOH (1 mL). The resulting dispersion was heated at 80 °C until complete dissolution of the solids and then cooled to room temperature. As per the standard preparation, solution B was poured in solution A under turboemulsification and further homogeneized for 30 s. The thus obtained dispersion became turbid a few seconds after the addition and was directly centrifuged (4500 rpm, 2 min) providing 272 mg of dried solid. No further purification nor size selection steps are performed.

### UV–Vis Absorption (ABS), Photoluminescence (PL), and Absolute PL Quantum Yield (QY) Measurements

The UV–visible absorption spectra were recorded using a Jasco V-570 UV–vis-NIR absorption spectrophotometer. Data were collected at 200 nm/min scanning speed. The PL spectra of all samples were measured on a Jasco FP-6200 fluorescence spectrofluorometer in a 90° geometry. The samples were excited with 365 nm wavelength (continuous light xenon lamp, Xe900). The excitation slit width was set at 5 nm. The detection slit width was set at 5 nm. The spectra were recorded with 1 nm steps and a scanning speed of 250 nm/min. The PL spectra were collected over a 375–600 nm spectral range. All samples were prepared by diluting NBs dispersions in toluene (0.1 mg/mL) in a 1 cm path length quartz cuvette. The PLQYs were measured using a FLS920 Edinburgh Instruments spectrofluorometer equipped with an integrating sphere. The optical density of the NBs solution was about 0.2 at the excitation wavelength of 405 nm (3.06 eV, obtained with picosecond laser diodes GaN, Picoquant LDH-P series, 70 ps pulses).

### Transmission Electron Microscopy (TEM) Characterization

Imaging was performed on a JEOL JEM 2100 Plus and JEOL JEM 2200FS, both operating at 200 kV. TEM samples were prepared by drop casting a toluene NCs dispersion (0.1 mg/mL) onto an ultrathin lacey carbon TEM grid. High resolution imaging was performed in parallel illumination mode using a CMOS Gatan RIO camera.

### Powder X-ray Diffraction (PXRD) Characterization

The analyses were performed using a benchtop Rigaku MiniFlex 600 operating at 45 kV and 40 mA equipped with a copper radiation source (Kα Cu 1.54 Å). PXRD samples were prepared by drop-casting a concentrated NC toluene dispersion (50 mg/mL) onto glass slides. Diffractograms were collected in a laboratory atmosphere and room temperature from 10° to 45° with steps of 0.02 and 0.2 deg/min speed.

### Inductively Coupled Plasma (ICP-OES)

The samples concentrations of Pb^2+^ and Cs^+^ ions were detected by an inductively coupled plasma-optical emission spectrophotometer (ICP-OES OPTIMA 7000 DV PerkinElmer). Each sample was digested in multiwave 5000 (Anton Paar) by adding nitric acid (HNO_3_ 65%) and hydrogen peroxide (H_2_O_2_ 30%) in a 8:2 volume ratio. This process was set in a closed system to reduce risk of contamination. The system is programmed to reach a power of 1000 W to ramp the detected temperature up to 220 °C. Then, the instrument keeps the vessel at 220 °C for 30 min. Digested samples were diluted with 10 mL of MQ water. After centrifuging and diluting (1:2), samples were ready to be analyzed by ICP-OES. Certified standard reference materials of Pb 1000 mg/L and of Cs 1000 mg/L (PerkinElmer) were used for calibration and quality control. The operating parameters of the ICP-OES instrument were set up using emission line at 220.353 nm for Pb and 455.531 nm for Cs in Axial View, and sample solutions were measured in triplicate. The detection limits for Pb and Cs are 0.01 mg/L.

## Results and Discussion

### Comparison of Magnetic Bar Stirring and Turboemulsification

The wet synthesis of colloidal NCs is plagued by concentration profiles, becoming progressively more relevant when increasing the reaction volume. Indeed, while working with rapid injection techniques at the proof-of-concept scale of 10–100 mL of solution and targeting samples of a few mg of dry NCs, high-speed stirring with either a magnetic stirring bar or overhead stirring is sufficient to handle concentration profiles. As we show, while moving to the liter scale, such methods are no longer appropriate. Turboemulsification represents one of the most popular industrial solutions for rapid and thorough mixing, particularly for microheterogeneous colloids like emulsions and dispersions. The Rotor-Stator Homogenizer Ultra Turrax (see Figure S1 for details) is a very popular and efficient choice.^37^

We were surprised to find out that turboemulsification was new to the synthesis of colloidal perovskites, materials almost universally produced using swift injection techniques where rapid and efficient mixing are paramount to ensure quality and reproducibility.

**Figure 1:**
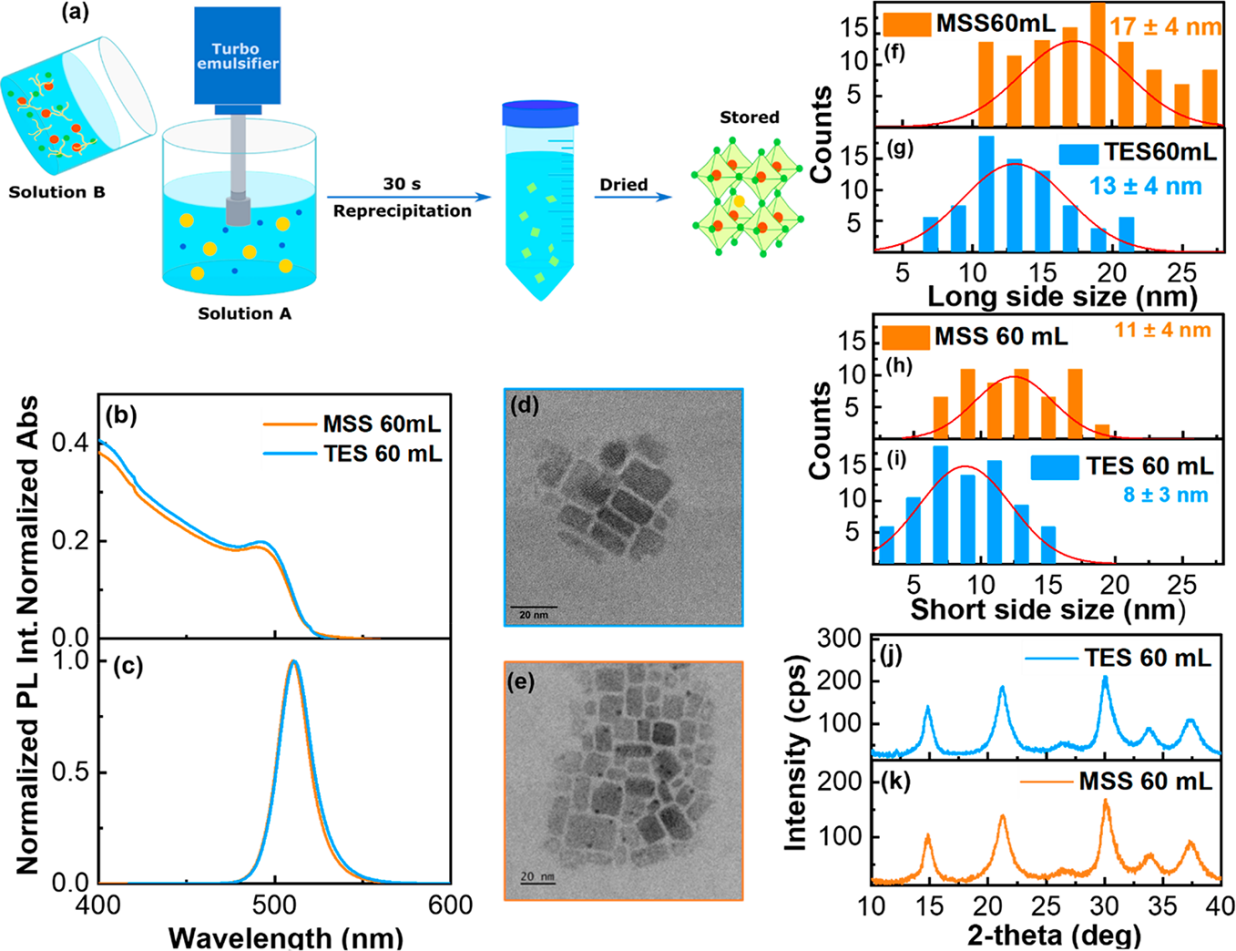
(a) Schematic representation of LHP-NCs: rapid injection of solution B (PbBr_2_ and TBAB dissolved in a OAm/PA 1:1 mol ratio mixture with iPrOH) into solution A (cesium propionate dissolved in Hept/iPrOH 2:1 vol ratio) under efficient turboemulsification; precipitation of CsPbBr_3_ NCs triggered by iPrOH; isolation by centrifugation and drying. Superimposed normalized absorption (b) and fluorescence (c) spectra of CsPbBr_3_ nanocrystals prepared with turboemulsifier (light blue) and magnetic stirring (orange). HRTEM images of (d) turboemulsified and (e) magnetic stirred samples. Nanocrystals (f, g) long side and (h, i) short side distribution evaluated from TEM for TES (light blue) and MSS (orange) with Gaussian fitting. (j, k) PXRD diffractograms of TES (light blue) and MSS (orange).

We first validated the use of turboemulsification in place of magnetic bar stirring in two identical reactions carried out on a typical laboratory scale of 60 mL reaction volume. With respect to the Akkerman protocol,^31^ we used OAm as the amine ligand. This choice enabled the preparation of stable colloidal dispersions, more suitable for detailed optical and structural characterization. We also added tetrabutylammonium bromide (TBAB, stoichiometric with PbBr_2_) to grow the target phase in the presence of excess bromide, according to recent results showing that such conditions help reducing the defectivity.^38−40^ The choice of the bulky tetrabutylammonium cation was made to suppress any competitivity with Cs^+^ in the incorporation in the NCs. The synthesis, described in detail in Experimental Section and schematized in Figure 1(a), requires pouring of a PbBr_2_/TBAB solution in an OAm/PA 1:1 mol ratio diluted with iPrOH into a solution of cesium propionate in a Hept/iPrOH 2:1 volume ratio mixture. During the whole addition and for an additional 30 s, the reaction requires thorough mixing. We prepared two distinct samples: one by mixing with the turboemulsifier (TES sample) and a control one using magnetic bar stirring at 1000 rpm (MSS sample). In both cases, the procedure leads to the formation of a clear yellow, strongly fluorescent solution. The addition of excess iPrOH triggers the precipitation of the NCs, that can be recovered by centrifugation. The MSS control experiment produced 97 mg of solid NCs, while the turboemulsified one gave 120 mg (64% and 78% yield, respectively, calculated with respect of Cs^+^ limiting reagent). The net amount of CsPbBr_3_ with respect of the organic ligand was estimated by thermogravimetric analysis (Figure S7). The two samples have indistinguishable optical and structural features and are both easily dispersible in toluene, thus validating the turboemulsification approach, as discussed ahead.

Interestingly, both samples, when redispersed in good solvents like toluene, show similar evolution of optical and structural features with time. The absorption spectrum of the as prepared solution of NPs shows a structured profile (ranging from 454 to 486 nm, corresponding to 2.55 eV - 2.73 eV, Figure S2(a)) that, according to TEM (Figure S2(d)), finds correspondence to NPs having different dimensions. Figure S2(b) and (c) show the second derivative of the spectrum and the assignment of the different zeroes to NPs having different degrees of confinement of the exciton, hypothesized from a literature quantitative equation.^38^ The TEM image, the statistical analysis (Figure S2(d–f)), and dynamic light scattering (DLS) analysis (Figure S4(a)) confirm the presence of the species identified in the UV–Vis absorption profiles. While in solution (with a kinetics that depends on the solvent employed, Figure S3(a–f)), they spontaneously evolve to the NBs morphology characterized by an aspect ratio that differs from 1 (Figure S4(b)). All details about this evolution process in solution are described in the SI. In regard to the method of preparation, the NPs are completely stabilized after 24 h of stirring at RT. Figure 1 shows that the materials prepared with MSSs and TESs on a 60 mL scale have comparable characteristics in terms of photophysical properties, shape, dimensions, and crystalline phase (Table S2). The samples exhibit a superimposable absorption profile with a peak at 496 nm and PL centered at 510 nm (Figure 1(b, c)). The full-width at half-maximum (FWHM) is also compatible and so the absolute photoluminescence quantum yield (PLQY, for both around 65%). The TEM images (Figure 1(d, e)) and the corresponding statistical analysis of dimensions (Figure 1(f–i)) reveal that for the TES sample, the long side is around 13 nm ± 4 nm and the short one around 8 nm ± 3 nm (leading to an average aspect ratio higher than 1, for TES 1.6 ± 0.4 and MSS 1.4 ± 0.3, Figure S6(g)). Values comparable within the experimental error are obtained for MSS sample. Considering random deposition of the NCs on every side, thickness can be estimated between 8 and 13 nm. The NBs have dimensions larger than the exciton Bohr radius for CsPbBr_3_ (3.5 nm),^16^ thus explaining the bulk like optical properties we observed. In terms of the specific crystalline structure, the PXRD data we acquired (Figure 1(j,k)) do not allow an unambiguous assignment. The NCs are small, which impacts on the line width. Also, the spectra are noisy due to the presence of amorphous material (mainly the ligand shell and excess ligand). Based on previous literature reports on low dimensional crystals with the same composition, we could have either a cubic or an orthorhombic phase, or even a mixture of the two.^39^ We did not observe batch-to-batch variations in the patterns.

Both morphology and crystalline structure are consistent with other low temperature protocols and ligand-rich conditions, that usually favor the formation of nanoplatelets over nanocubes.^40,41^ Our procedure does not involve any purification nor size selection.

### Effect of Scaling Up

**Figure 2:**
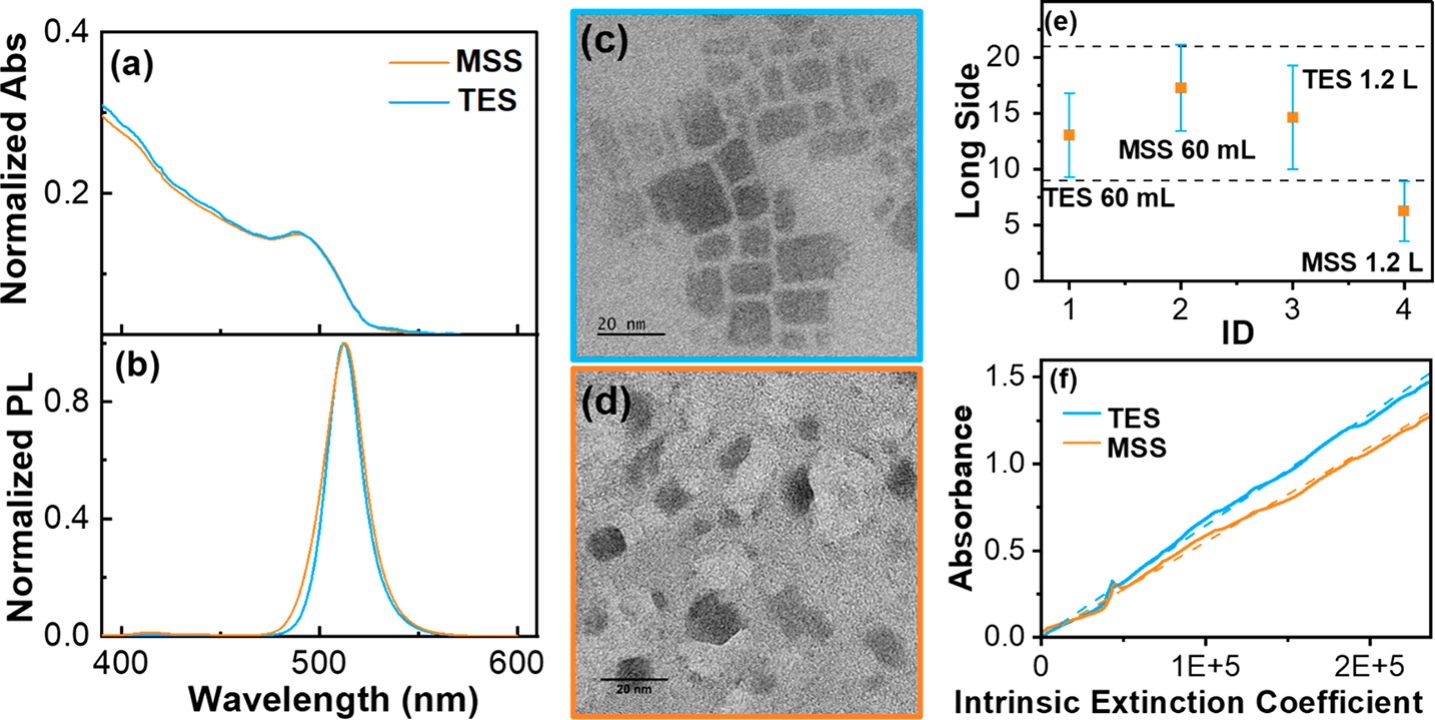
Normalized (a) absorption spectra and (b) fluorescence spectra of perovskites nanocrystals inks obtained from 1200 mL batches with turboemulsifier homogenizer (light blue line) and traditional magnetic stirring (orange line). HRTEM images of samples obtained, respectively, with (c) turboemulsification and (d) magnetic stirring (e). Plot of the long side statistical mean values for TES and MSS at 60 and 1200 mL scales (50 NPs). (f) Absorbance at the specific wavelength (489 nm) as a function of the intrinsic extinction coefficient, calculated by De Roo et al.^42^ and their linear regressions for both TESs (light blue) and MESs (orange).

Having demonstrated the equivalence of magnetic stirring and turboemulsification while working on a standard lab scale, we linearly scaled both procedures up to a 1200 mL volume of the reaction mixture, without any other change in the protocol. In terms of quantity of isolated materials, the two procedures still gave comparable results: 2.8 g of dry nanocrystals under turboemulsification and 2.6 g with magnetic stirring. The quality of the two batches was dramatically different. Indeed, if the steady state optical properties of the two samples are still comparable (Figure 2(a, b), Table S2), the structural TEM analysis provides a very different scenario (Figure 2(c, d)). The NCs produced by TESs are indistinguishable from those produced at the small scale (Figure S6); conversely, the MSS sample is contaminated by large quantities of an amorphous phase, distinct from the target one (Figure 2(d)). The crystalline NPs are still present, albeit with a morphology distinct from NBs and more closely resembling flakes (Figure 2(d)). Figure 2(e) shows the comparison between the average value of the longer side for TES and MSS materials, highlighting the deviation from the results obtained with the latter with respect to the experiment performed on a lab scale. TEM suggests that, even though the amount of the materials we isolated in the two procedures was comparable, the actual NCs yield is higher in the case of the TES. Indeed, we compared the optical density of NC solutions having the same wt % concentration obtained with TES and MSS samples, and we obtained different optical densities. This can only be explained by assuming that the MSS sample is contaminated by material distinct from the CsPbBr_3_ target phase. The difference between the two samples can be quantitatively correlated with the purity using a method developed by De Roo et al.^42^ The absorbance and concentration of NCs of the CsPbBr_3_ phase can be correlated through the introduction of intrinsic extinction coefficient. The authors calculated such parameters for a wide range of wavelengths under appropriate conditions.

We plotted the absorbance at a given concentration of the two solutions as a function of the intrinsic extinction coefficient (Figure 2(f)). The data sets (from 320 to 550 nm) can be satisfactorily fitted with a straight line (Table S3). The resulting slope, that is demonstrated to be proportional to the effective NCs concentration in solution, is distinguishably higher in the case of the TES sample, even considering the experimental error. This behavior confirms that the actual concentration of NBs in the MSS sample is smaller than in the case of the TES one. The deviation from linearity between 458 and 512 nm is related to the NBs morphology, contributing more significantly to absorption in that region with respect to the nanocubes used to define intrinsic extinction coefficients. We further compared the two samples by TGA and ICP-OES to evaluate the ligand concentration and to characterize the Cs/Pb ratio (Figure S8, Table S4).

Since the two samples gave similar results, the amorphous material contaminating the TES sample could consist of either oleylammonium-rich lead bromide clusters or very small Cs_4_PbBr_6_ 0D nonluminescent clusters. In regard to the structure and compositions, both materials are undesired byproducts (Figure S9). Quite clearly the very efficient and rapid mixing provided using the turboemulsifier avoids their formation.

### Reproducibility and Reliability of the Procedure

**Figure 3:**
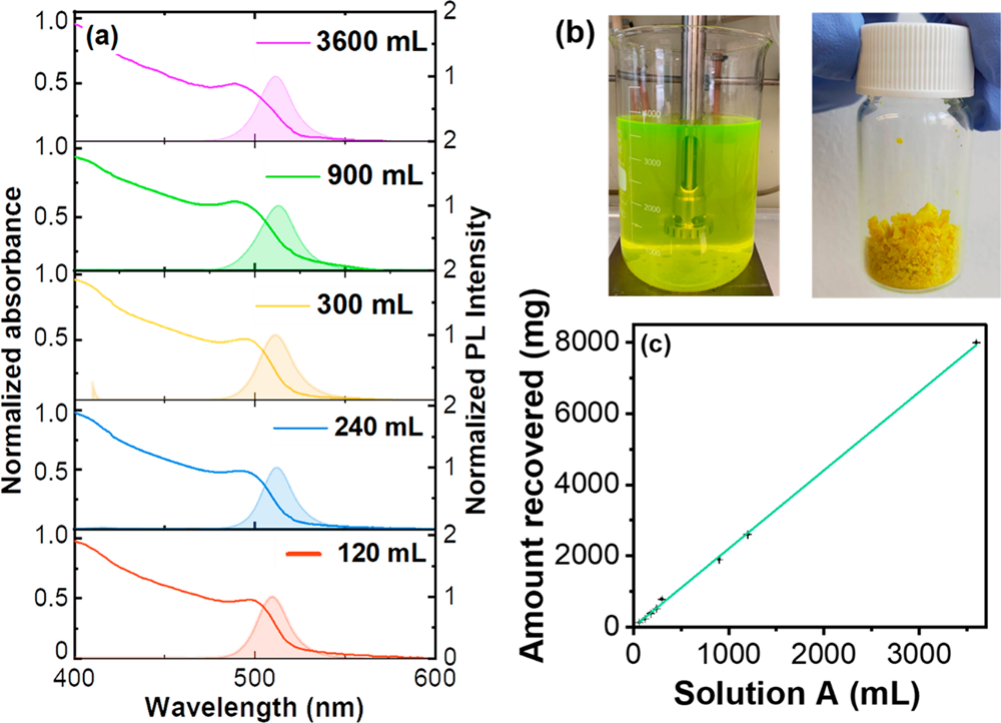
(a) Normalized absorbance (only curves) and photoluminescence spectra (filled area curves) of five samples of different scales that show comparable features. (b) Photographs of the obtained 3.6 L raw reaction mixture (on the left) and 8 g of sample in a 20 mL transparent vial (on the right). (c) Linear regression of the dried recovered amount of NCs as a function of the volume of solution A.

Reproducibility and reliability frequently affect the synthesis of inorganic and hybrid perovskites. While moving from the lab to the fab environment, processes must be robust and predictable over a wide range of scales. We thus tested the behavior of the TES protocol at increasing reaction volumes of 120, 240, 300, 900, 1200, and 3600 mL. Figure 3(a) shows the normalized absorption and fluorescence spectra of all the different samples we obtained. Table 1 shows the corresponding optical parameters (fluorescence maximum, FWHM, first absorption peak), together with the amount of recovered NCs. Also the PLQY ranges between 58% and 69%, remaining comparable within the error value.

**Table 1:** Every Entry Represents a Sample for Which Are Reported the Volume of Solution A Employed, Dried Recovered Amount of Material, Absorption Peak, Maximum of Photoluminescence, and Corresponding Full Width at Half Maximum^a^

ID	Scale (mL)	Weight (g)	ABS Peak (nm)	PL Max (nm)	FWHM (nm)
1	60	0.120	498	510	21
2	120	0.230	498	508	26
3	240	0.510	496	510	20
4	300	0.780	497	510	20
5	900	1.9	495	512	25
6	1200	2.6	497	508	24
7	3600	8	495	511	25

a Data are comparable at every scale.

The TEM analysis shows comparable results while scaling up (Figure S10). In terms of optical properties, we observed very small batch-to-batch variations that could be relevant for some specific applications like LEDs but that have little (if any) impact on others like plastic scintillators, LSCs, and solar cells. As already stated, our method does not include any purification nor size selection procedure. This was a deliberate decision to keep the method simple and to ensure the full recycling of all chemicals we employed.

The data shown in Table 1 are particularly meaningful in view of standardized production: the batch size and the amount of recovered material are linearly correlated. (Figure 3(c), Table S5). We limited the size of the reaction to a 3.6 L volume only because the largest beaker we could find had a volume of 4.5 L, and we had to allow for the addition of the iPrOH, necessary to trigger precipitation of the NPs (Figure 3(b)). We finally tested the generality of the protocol by using lead halogenides other than bromide. We succeeded in the syntheses of CsPbCl_3_ and mixed CsPbBr_*x*_Cl_3–*x*_ (Section S13, Figure S11), even if chloride and bromide anions have a different reactivity and kinetics in the CsPbX_3_ formation process. We tested the procedure with a shorter amine as well (BuAm, Section S14, Figure S12) without significant changes in the quality of the outcome.

### Recovery and Recycle of Excess Reagents and Solvents

The wet syntheses of NCs commonly require relatively dilute conditions and excess reagents (lead halogenide and OAm in our case) and can be affected by the formation of byproducts. Generally, the amount of wastes produced outnumbers the product by orders of magnitude. In the case of lead halide perovskites, the issue of wastes is particularly severe because of the contamination with non-negligible amount of toxic lead compounds. While there are ongoing efforts to recover the lead from spent perovskite-containing devices, to the best of our knowledge, the literature does not report real strategies for the recycle of the wastes produced during the synthesis, but only a few preliminary and overlooked works.^43^

The procedure we developed has the advantage of being based on the use of solvents having low boiling point and high volatility, thus making recovery by distillation easy and energy efficient. We explored the possibility of recycling not only the volatile solvents but the nonvolatile residue as well.

We tested the recovery procedure on a reaction batch of 240 mL. After the centrifugation procedure, we recovered 510 mg of colloidal NCs and around 360 mL of discarded supernatant.

After evaporation under reduced pressure, we obtained 354 mL of Hept/iPrOH mixture 1:1.3 in volume ratio (for details on the determination of the mixture composition, see Section S15) and 16 mL (15.2 g) of a nonvolatile mixture of unreacted species. GC-MS analysis detected no contaminant in the recovered solvent mixture, which could then be reused for further synthetic purposes. The in solution ^1^H NMR analysis of the nonvolatile residue shows a mixture of OAm, PA, and TBAB, in relative concentration corresponding with the feeding ratio (Figure S13). Other than precipitation of the target CsPbBr_3_ phase, no other chemical modification happens in the sample.

We also measured the Cs^+^ and Pb^2+^ concentrations at 0.04 and 2.25 wt %, respectively, by X-ray fluorescence (XRF) combined with ICP-OES and TGA (Figure S14(a, b), Table S7).

This information is crucial in view of recycling as Cs^+^ is the limiting reagent, and the Cs/Pb ratio influences the NC composition and morphology. We thus used the nonvolatile residue for the synthesis of a new batch of NCs, adding only the minimum amount of fresh reagents necessary to obtain the correct stoichiometry (the detailed procedure is described in the Experimental Section and in Section S16 of the SI). After few seconds from the addition of solution B into solution A, the NCs precipitate by themselves without the addition of iPrOH. The total amount of material recovered, without the addition of iPrOH, is 272 mg, in good agreement with the 264 mg predicted by the linear relationship shown in Figure 3(c).

**Figure 4:**
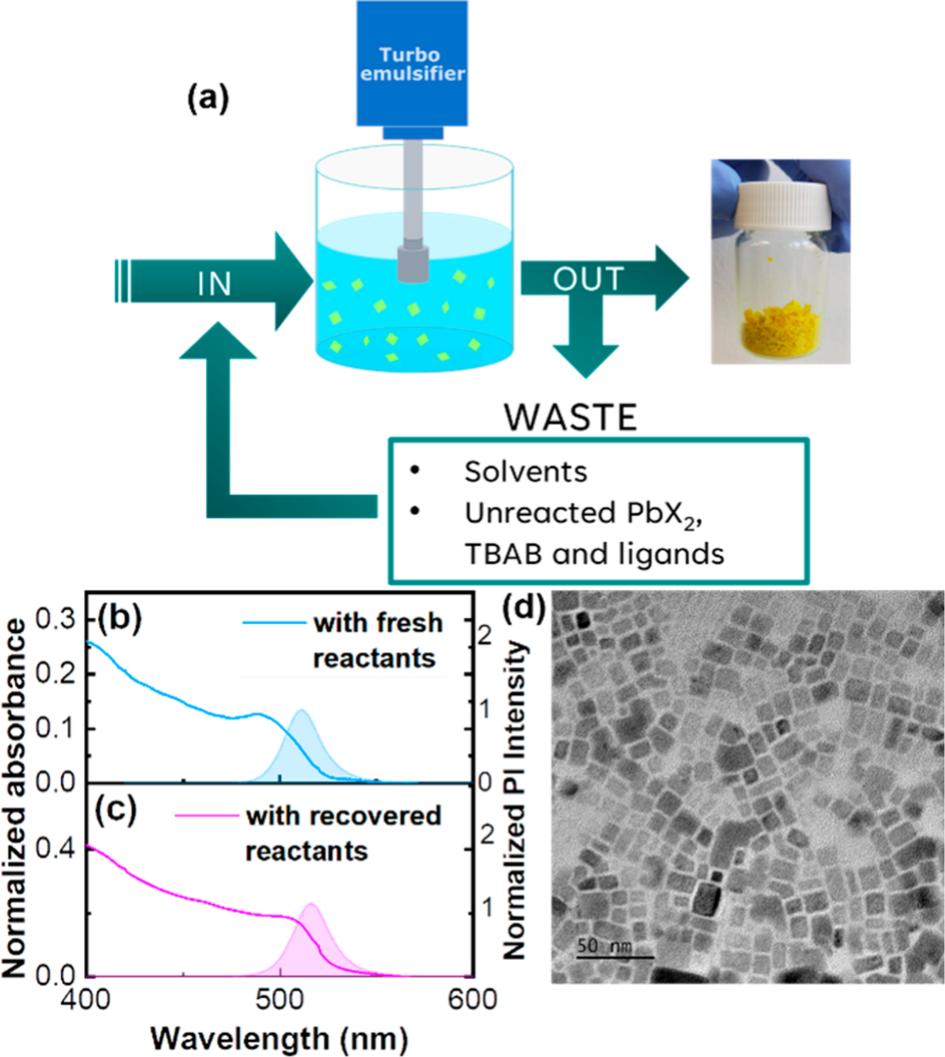
(a) Schematic representation of the “circular” synthetic process that takes advantage of wastes reuse. Normalized absorption (full line) and photoluminescence (filled area curve) spectra of nanocrystals synthesized with fresh reactants (b) and recovered precursors (c). (d) HRTEM image of a CsPbBr_3_ sample prepared with recovered wastes.

Figure 4(b–d) compares the optical and morphological characteristics of the NCs obtained from recycled reagents with those of the sample made with fresh ones. The features of the samples obtained with recycled materials have some meaningful differences that can be explained while considering that the process involves a seeded precipitation. The addition of iPrOH to the original reaction mixture triggers the precipitation of most but not all NCs thus formed. This can be clearly deduced from the fact that the supernatant we obtain after the centrifugation step is still yellow and luminescent. While preparing the NCs using the recycled reagents, such smaller NCs of the CsPbBr_3_ phase are already present in the Pb/oleyl amine solution and have an impact on the outcome of the procedure. First, the upcycled materials do not require the aging time that is conversely necessary for the stabilization of the optical properties of the NCs prepared using fresh starting materials. Moreover, the morphology also changes: the NCs are bigger and possess an aspect ratio (1.5 ± 0.6) that is slightly different from the one of the materials prepared with fresh reagents (1.6 ± 0.4). The Supporting Information reports a statistical analysis of the dimensions (Figure S15(a,b)). While there is a noticeable difference in the absorption profile of recycled vs pristine NCs, the luminescence efficiency (PLQY 65%), most crucial for application, remains essentially unchanged.

In principle, the supernatant of this second reaction could again be recycled. We did not test more than one recycle yet.

## Conclusion

We developed a synthetic procedure for CsPbBr_3_ NCs that is simple, reliable, and scalable up to the unprecedented scale of 8 g of dry material in a single preparation. The key feature of the method is the use of turboemulsification as the mixing method during the rapid injection step. The detailed, multidisciplinary optical, PXRD, and TEM characterizations show high quality and very remarkable reproducibility over 2 orders of magnitude variation of the reaction volume. The control experiment performed using standard magnetic bar stirring shows progressive contamination of the recovered material with nonemissive, amorphous byproducts as the scale increases. Our improved procedure scales linearly from milliliters to liters changes in the reaction volume, thus being fully predictive in a standardized production environment. We also fully characterized the wastes produced during the synthesis, devising a protocol for their complete recycle. Our results represent an important step beyond for lead halide perovskite industrialization from the standpoint of efficiency, scalability, and waste reduction thus contributing significantly to the level of acceptance of this technology in established and emerging fields like solar cells and plastic scintillators.

## Supporting Information Available

Detailed description of the turboemulsification setup. Details on the evolution of the nanobrick structures and optical properties in different solvents. NMR characterizations of the nanomaterials. Thermogravimetric analyses. PXRD and TEM (including statistical analyses of dimensions and aspect ratio) of materials obtained working on the different reaction scales. Time resolved photoluminescence. (PDF)
