# Supplementary material for: Multigram-Scale Synthesis of Luminescent Cesium Lead Halide Perovskite Nanobricks for Plastic Scintillators
Source: ACS Appl Nano Mater. 2023 May 31;6(11):9436–43. doi: 10.1021/acsanm.3c01146 (PMC10262149; doi:10.1021/acsanm.3c01146)
Supplement: Supplementary file 1 — an3c01146_si_001.pdf [file an3c01146_si_001.pdf]

Supporting Information for:

Multigram-Scale Synthesis of Luminescent Cesium Lead Halide Perovskite Nanobricks for Plastic Scintillators

Sara Mecca,[a] Francesca Pallini,[a] Valerio Pinchetti,[a] Andrea Erroi,[a] Alice Fappani,[a] Francesca Rossi,[b] Sara Mattiello,[a] Giovanni Maria Vanacore,[a] Sergio Brovelli[a] and Luca Beverina[a,*]

[a]University of Milano-Bicocca, Department of Materials Science, via R. Cozzi 55, I-20126 Milan, Italy
[b]IMEM-CNR Institute, Parco Area delle Scienze 37/A, 43124 Parma (IT)
*luca.beverina@unimib.it

TABLE OF CONTENT

S1 - HIGH SHEAR TURBO EMULSIFIER HOMOGENIZER S2
S2 – METASTABLE OUTPUT DESCRIPTION S3
S3 –CHARACTERIZATION OF MATERIAL EVOLUTION: DIMENSIONS AND OPTICAL PROPERTIES IN SOLUTION S4
S4 –SYNTHESIS OF $CsPbBr_3$ NANOCRYSTALS WITH MAGNETIC STIRRING (1.2 L SCALE) S6
S5 – TIME RESOLVED PL EXPERIMENTS COMPARISON (ON EVOLVED TES AND MSS 1.2 L) S6
S6 -STATISTICAL ANALYSIS ON DIMENSIONS AND ASPECT RATIO S7
S7 – TGA OF TES AND MSS (60 mL SCALE) S8
S8 – CONCENTRATION OF THE $CsPbBr_3$ NCs DISPERSIONS S9
S9 – TGA AND ICP-OES OF TES AND MSS (1.2 L SCALE) S10
S10 – PXRD OF TES AND MSS SAMPLES (1.2 L BATCH) S11
S11 – TEM IMAGES OF SAMPLES PREPARED AT DIFFERENT VOLUME SCALE S12
S12 - LINEAR MODEL FOR AMOUNT RECOVERED IN FUNCTION OF SYNTHESIS VOLUME S13
S13 – SYNTHESIS AND STEADY STATE OPTICAL CHARACTERIZATION OF $CsPbCl_3$ AND $CsPbBr_xCl_{3-x}$ NANOCRYSTALS S14
S14 –$CsPbBr_3$ AND $CsPbCl_3$ WITH BUTYLAMINE (BuAM) LIGAND S16
S15 – RECOVERY: SUPERNATANT CHARACTERIZATION S17
S16 –SYNTHESIS WITH RECOVERED REACTANTS S19

S1 - HIGH SHEAR TURBO EMULSIFIER HOMOGENIZER

The high shear turbo emulsifier employed consists in a motor group (IKA ULTRA-TURRAX 25 (A) or 50 (B) DIGITAL) and a dispersing tool (S25N-25G (C) or S50N-G45M (D), whose characteristics are described in table S1).

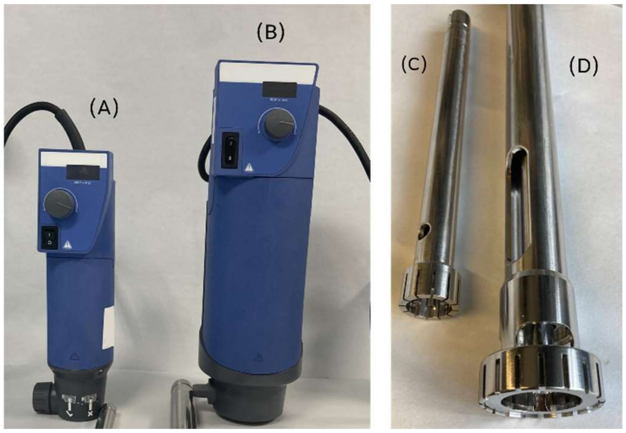

Figure S1. The motor group IKA ULTRA-TURRAX DIGITAL 25 (A) and 50 (B) and the respective dispersing tools S25N-25G (C) and S250N-G45M (D).

Table S1. Characteristics of the two dispersing tools employed.

	Code	Stator	Rotor	Tmax	Fineness dispersions	Fineness emulsions	Max rotations	V range
C	S25N–25G	25 mm	17 mm	180 °C	15-50 µm	1-10 µm	24k RPM	50-2000 mL
D	S50N-G45M	45 mm	40.5 mm	180 °C	25-50 µm	5-20 µm	10k RPM	500-15000 mL

S2 – METASTABLE OUTPUT DESCRIPTION

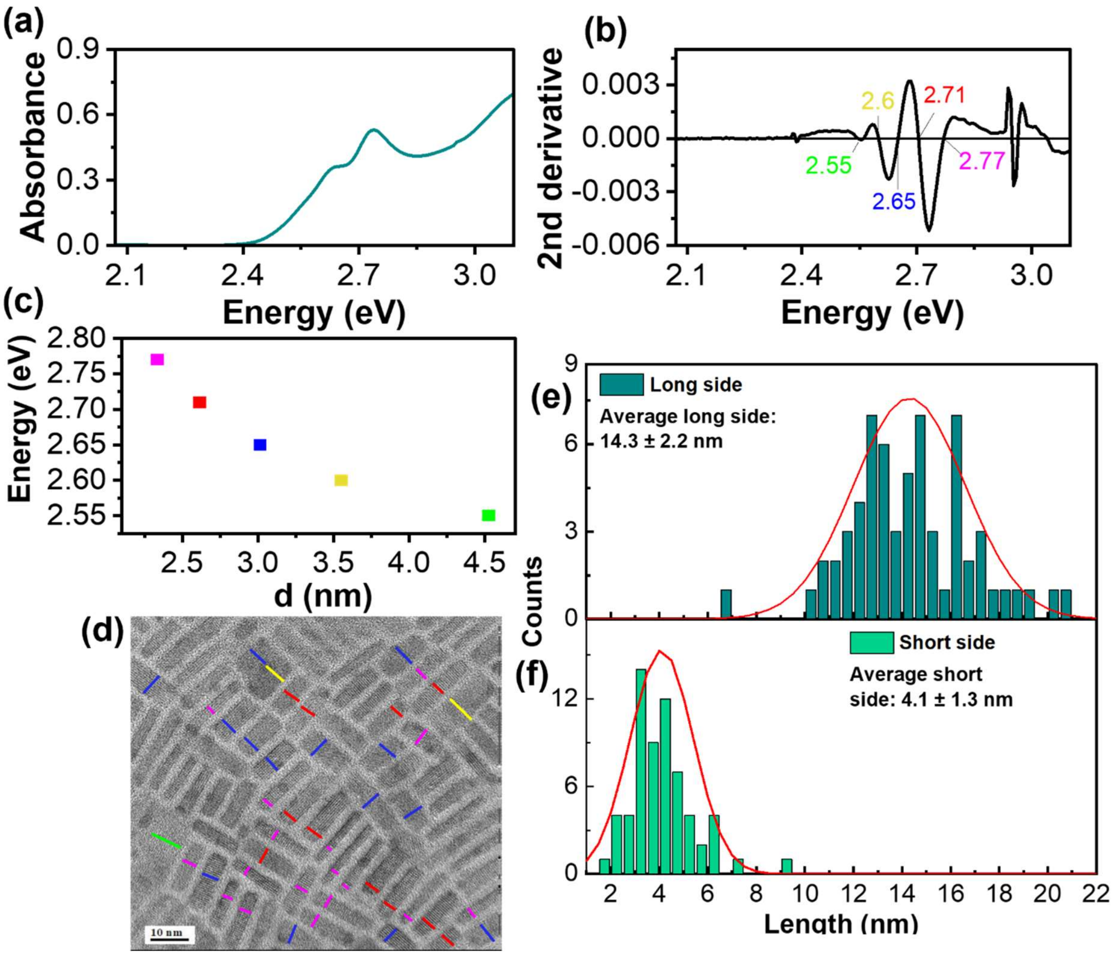

Figure S2. Metastable samples characterization. (a) Absorption spectrum and (b) its second derivative for the pristine sample just redispersed in toluene at 0.1 mg/mL. For the latter, the intercepts with the X axis are reported and hint at the energy gaps associated with the various contributions of crystals of different size (identified by different colors). (c) Graph of the correspondence between the derived energy gaps and the calculated dimensions of the NCs. These values are derived from Diliberto et al. theoretical relation[1]. (d) High resolution transmission electron microscopy (HRTEM) image of the pristine NCs and the corresponding distributions of dimension of (e) long and (f) short side, fitted with a gaussian curve, both with the average value and the standard deviation.
(70 NPs). In the former, the short side is highlighted with differently colored bars: < 4 nm pink, between 4.01 and 5 nm red, between 5.01 and 7 nm blue, between 7.01 and 9 nm yellow, above 9 nm green.

S3 –CHARACTERIZATION OF MATERIAL EVOLUTION: DIMENSIONS AND OPTICAL PROPERTIES IN SOLUTION

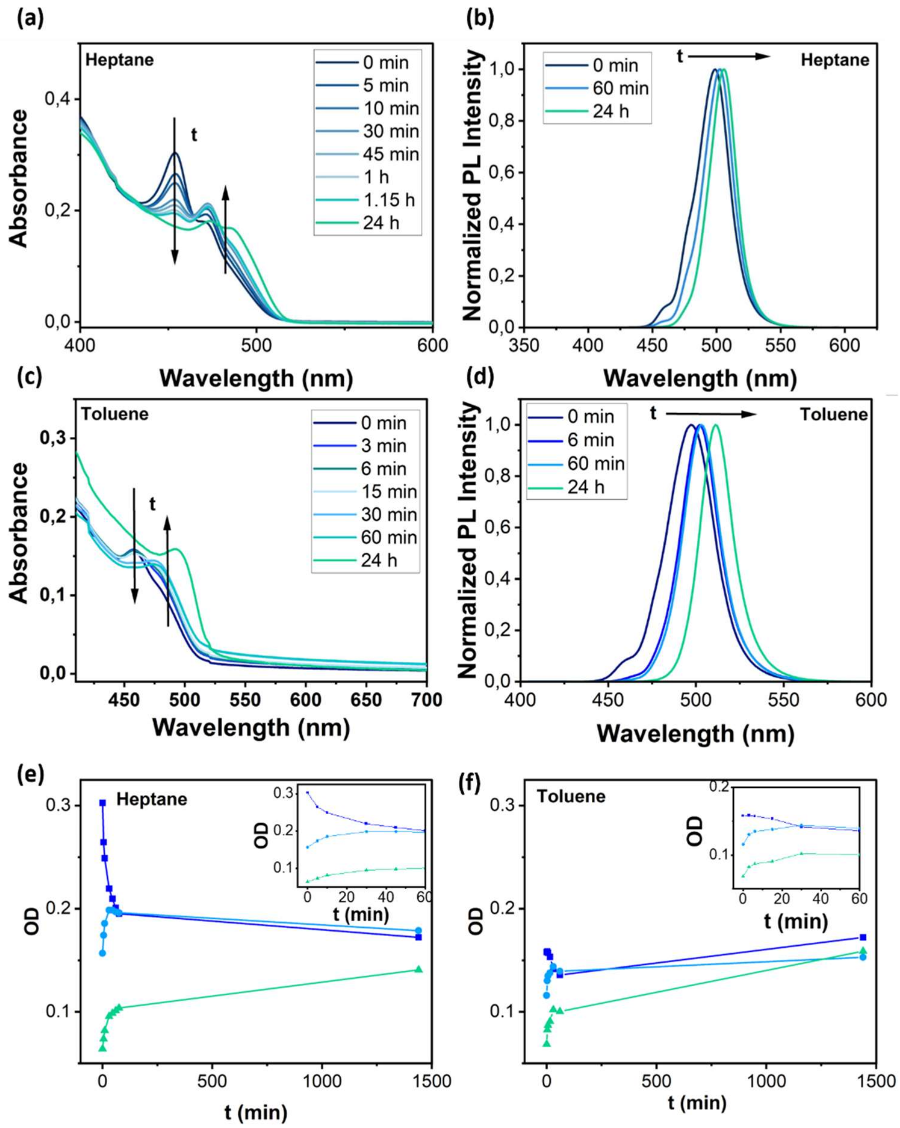

Figure S3. Optical characterization of in solution evolution of $CsPbBr_3$ output material. Absorption spectra over time (from the as prepared solution to 24 hours under stirring at RT) of a 0.1 mg/mL sample in heptane (a) and toluene (c) and (b-d) the respective normalized photoluminescence spectra as prepared, after 1 h and after 24 h. Absorption values trend at different wavelengths (458, 476 and 493 nm) as function of time for $CsPbBr_3$ NCs dispersed in toluene (e) and (f) heptane (0.1 mg/mL). Zoom from 0 to 60 minutes in the inset.

Samples preparation. The dried solid state $CsPbBr_3$ NCs were dispersed under magnetic stirring in the selected solvent (toluene or heptane) at a concentration of 10 mg/mL and after 30 minutes of homogenization, 200 µL were picked up with a Hamilton glass syringe (range 10-250 µL) and diluted to 2 mL directly in a 1 cm path quartz cuvette. The first spectrum, indicated as 0 min, was collected as dilution occurred. The solution was then kept under magnetic stirring inside the spectrophotometer and the further absorption spectra were recorded at indicated times. Photoluminescence spectra were recorded at longer time intervals in the adjacent spectrofluorometer. The UV–visible absorption spectra were recorded using a Jasco V-570 UV-Vis-NIR absorption spectrophotometer. Data were collected with

200 nm/min scanning speed. The PL spectra of all samples were measured on a Jasco FP-6200 fluorescence spectrofluorometer in a 90° geometry. The samples were excited with 365 nm output of the continuous xenon lamp (Xe900). The excitation slit width was set at 5 nm, the detection slit width was set at 5 nm, and the spectra were recorded with 1 nm steps and a scanning speed of 250 nm/min. The PL spectra were collected over a 375–600 nm spectral range. It is possible to notice that the solvent that solubilized more the ligands shell (toluene), fastens from the beginning the evolution process.

Table S2. Every entry represents a type of sample (TES – turbo-emulsified sample – and MSS – magnetically stirred sample) for which are reported the volume of solution employed, the dried recovered amount of material, the absorption edge, the maximum of photoluminescence, the corresponding full width at half maximum and the aspect ratio with its standard deviation. The data are comparable at every scale.

Entry	Volume, mL	Output, mg	Absorption peak, nm	PL maximum, nm	FWHM, nm	Aspect ratio
TES	60	120	493	511	22	1.6 ± 0.4
	1200	2800	489	513	24	1.8 ± 0.8
MSS	60	97	490	510	21	1.4 ± 0.3
	1200	2700	491	512	20	1.4 ± 0.4

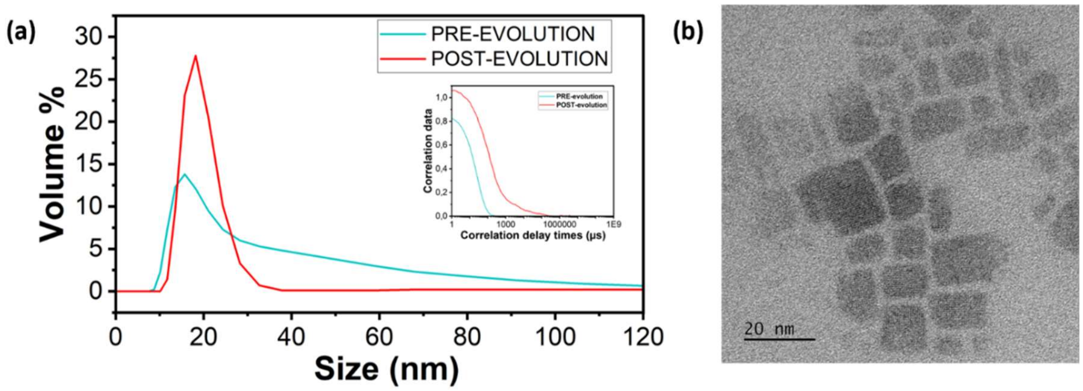

Figure S4. (a) Dynamic light scattering (DLS) analysis of the pristine (light blue curve) and evolved (red curve) sample. (b) TEM image of a $CsPbBr_3$ sample evolved in toluene at 0.1 mg/mL under stirring a 25 °C overnight and then deposited by drop casting directly on the TEM grid.

Dynamic light scattering (DLS) analysis were performed using Malvern instrument Nano-S, whose data processing software is Zetasizer. The refractive index (RI) of the material was indicated as 1.9[2] with absorption 0.0 at the laser probe wavelength (633 nm). Dispersant was toluene (RI 1.496). Samples were prepared dispersing the solid product in toluene under magnetic stirring at 10 mg/mL and then diluiting 75 μL of that solution to 1 mL of pure toluene (0.075 mg/mL) and measured as prepared (non-evolved sample, green line) and after 24h (evolved, red line) under magnetic stirring. Analysis have been performed on 1 mL of solution in a 1 cm quartz cuvette. Measurements have been collected at 25 °C (equilibration time: 30 seconds), positioning the laser focus in the centre of the cuvette. Three consecutive measurements have been performed on each sample (each measurement had 17 runs). When data met quality criteria, such measurement were averaged to obtain the final volume distribution through a distribution fit. Given that the instrument collects scattering data only at a fixed angle configuration (173°), the result doesn't consider anisotropy in samples. In this non-spherical case, the output value must be considered thus as the diameter of an approximate sphere around the nanobrick sample, resulting in a bigger value respect to TEM evaluation.

S4 –SYNTHESIS OF $CsPbBr_3$ NANOCRYSTALS WITH MAGNETIC STIRRING (1.2 L SCALE)

A solution containing the Cs^+ precursor (from here on defined as Solution A) is prepared by reacting Cs_2CO_3 (2 mmol, 650 mg) with propionic acid (2 mL, 26.6 mmol) and then diluting the resulting solution with 1200 mL of heptane/isopropanol 2:1 vol mixture. A solution containing the Pb^{2+} precursor (from here on defined as Solution B) is prepared by dissolving $PbBr_2$ (20 mmol, 7.34 g) and tetrabutylammonium bromide (20 mmol, 6.44 g) in a mixture of oleylamine (180 mmol, 59.2 mL), propionic acid (180 mmol, 13.4 mL) and isopropanol (13.4 mL) at 80 °C. After the complete dissolution of the precursors, the mixture is cooled to room temperature (no precipitation observed over a period of at least 8h). Solution A is put under magnetic stirring with magnetic bar (diameter 10 mm x length 60 mm) at 1000 RPM in a 2.5 L beaker, then solution B is swiftly poured identically to turbo-emulsified procedure. The mixture is further homogenized for 30 s to give a clear yellow solution. The latter is diluted with 600 mL of isopropanol, observing the formation of a fine precipitate that is collected by centrifugation at 4500 RPM for 2 min. The supernatant is recovered and recycled as described below, whilst the bright yellow solid is dried under reduced pressure till constant weight (2.8 g) and stored in a glove box under argon atmosphere. No further purification nor size selection steps are performed.

S5 – TIME RESOLVED PL EXPERIMENTS COMPARISON (ON EVOLVED TES AND MSS 1.2 L)

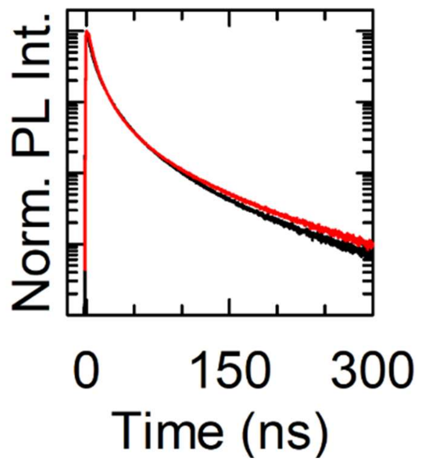

Figure S5. Time resolved PL decay of respectively of TES 1.2 L (red line) and MSS 1.2 L (black line) samples.

Time-Resolved PL Experiments. Spectroscopic studies were carried out on toluene solutions of NCs. For measurement, the samples were excited using a frequency tripled pulsed Nd:YAG laser at 3.49 eV with a 100 Hz repetition rate (pulse duration, 5 ns), and the emitted light was collected with a time-correlated single photon counting (TCSPC) (resolution better than the pulse duration). Measurements are performed on diluted NC dispersions, in a 45° geometry in order to minimize the self-absorption effects.

S6 -STATISTICAL ANALYSIS ON DIMENSIONS AND ASPECT RATIO

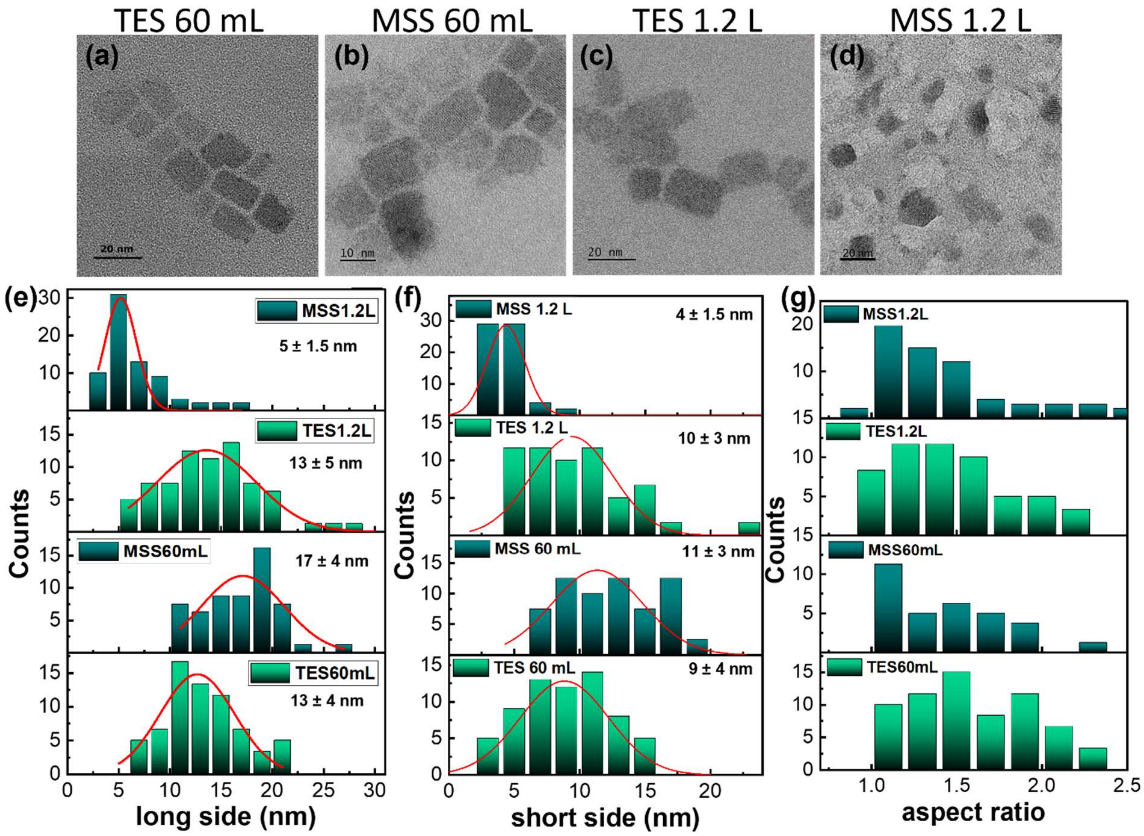

Figure S6. TEM images respectively of (a) TES on 60 mL scale, (b) MSS 60 mL, (c) TES 1.2 L, (d) MSS 1.2 and the statistical analysis of (e) long side, (f) short side with gaussian fitting and (g) aspect ratio of the aforementioned samples.

S7 – TGA OF TES AND MSS (60 mL SCALE)

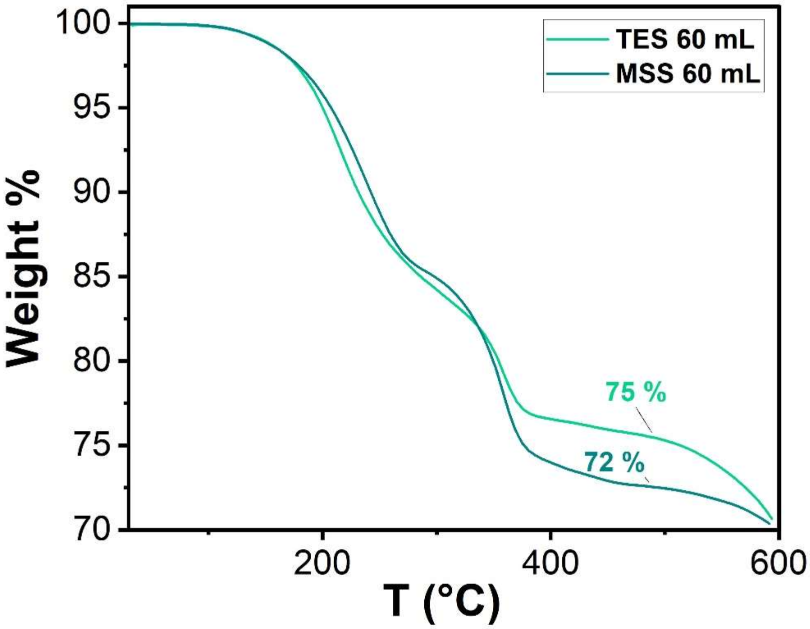

Figure S7. TGA analyses of TES (light green) and MSS (dark green) samples, obtained from the synthesis on 60 mL scale.

Thermogravimetric analysis (TGA) characterization. Measurements were carried out on TGA/DSC STARe System (Mettler TOLEDO). Solid state samples (around 15 mg) were directly put inside an alumina crucible. Method: from 30 °C to 600 °C, heating at 10 °C/min, under N_2 flow (50 mL/min).

S8 – CONCENTRATION OF THE $CsPbBr_3$ NCs DISPERSIONS

We adopted this procedure from a De Roo et al.[3] work, where the following equation was derived:

$$A(\lambda) = \frac{N_A V_{NC} L}{ln10V_0} c\mu_i(\lambda) = k(c)\, \mu_i(\lambda)$$

A sample absorbance at a given concentration; N_A Avogadro's number; V_{NC} NC unit cell volume; L cuvette length; V_0 solvent volume in nm^3; c NCs concentration; μ_i intrinsic extinction coefficient for each wavelength between 320-550 nm reported in ref [2]. The model based on a linear fit of the absorbance value in function of μ_i suggests that the angular coefficient ($k(c)$) is proportional to the effective material concentration in sample.

Samples preparation and data obtainment. Samples produced by both homogenization types with 1.2 L batches are weighted and dispersed directly in toluene at a nominal concentration of 1 mg/mL and stirred overnight. They are then diluted at weight concentrations of 0.125 mg/mL and absorption spectra are collected. Absorption values are plotted in function of intrinsic extinction coefficient between 320 and 550 nm with steps of 2 nm and a linear fitting is performed. The result is reported in Table S2.

Table S3. For the linear regression of both samples, the slope, the intercept and the R^2 are reported.

Sample	Slope	Intercept	R^2
**TES 1.2 L**	6.39E-9 ± 3E-11	0.006 ± 0.004	0.997
**MSS 1.2 L**	5.27E-9 ± 3E-11	0.029 ± 0.003	0.996

S9 – TGA AND ICP-OES OF TES AND MSS (1.2 L SCALE)

Details about TGA and ICP-OES sample preparation and analysis are reported before (respectively in Section S8 and in Methods).

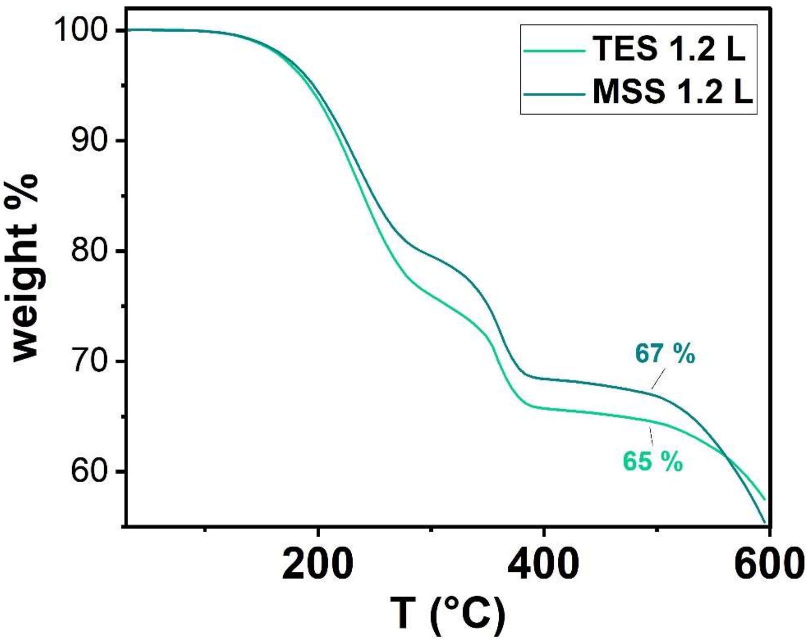

Figure S8. TGA analysis comparison of TES and MSS sample on 1.2 L batch scale.

Table S4. For each sample, the identification, the analyzed sample weight, the weight % of both cesium and lead from ICP-OES and their ratio are reported.

Sample	Weight	wt% Cs	wt% Pb	Pb/Cs
**TES 1.2 L**	112 mg	10.93	21.28	1.2
**MSS 1.2 L**	111 mg	11.6	22.27	1.2

Considering the different atomic weight of cesium and lead (respectively 132,9 g/mol and 207,2 g/mol), compatible with the ionic form, the Pb/Cs ratio of the two sample is comparable and around 1.2. This value is slightly higher than the stoichiometric expected ratio of 1. This is probably related to $[PbBr_4]^{2-}$ terminated surfaces that are abundant in high surface area samples as these nanobricks.

S10 – PXRD OF TES AND MSS SAMPLES (1.2 L BATCH)

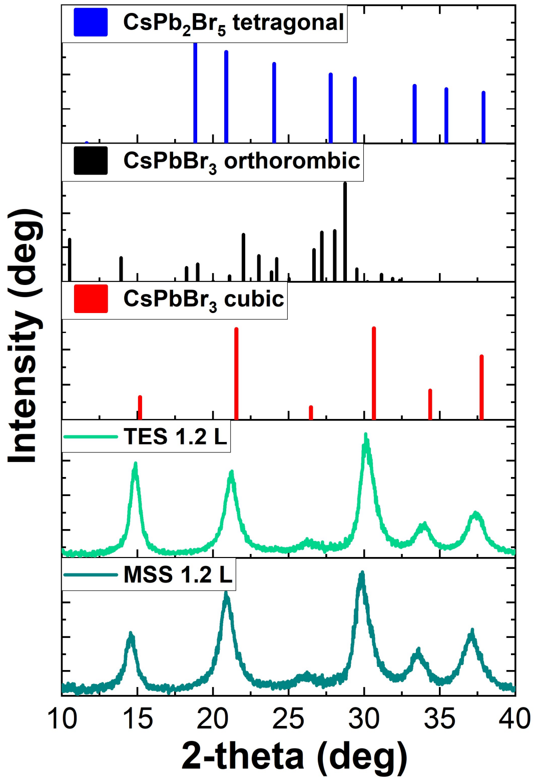

Figure S9. PXRD diffractograms of TES and MSS samples on 1.2 L scale respect to reference orthorhombic and cubic $CsPbBr_3$ (respectively PDF Card No.: 01-074-2251 and No.: 01-084-0464) and tetragonal $CsPb_2Br_5$ (PDF Card No.: 00-054-0753).

S11 – TEM IMAGES OF SAMPLES PREPARED AT DIFFERENT VOLUME SCALE

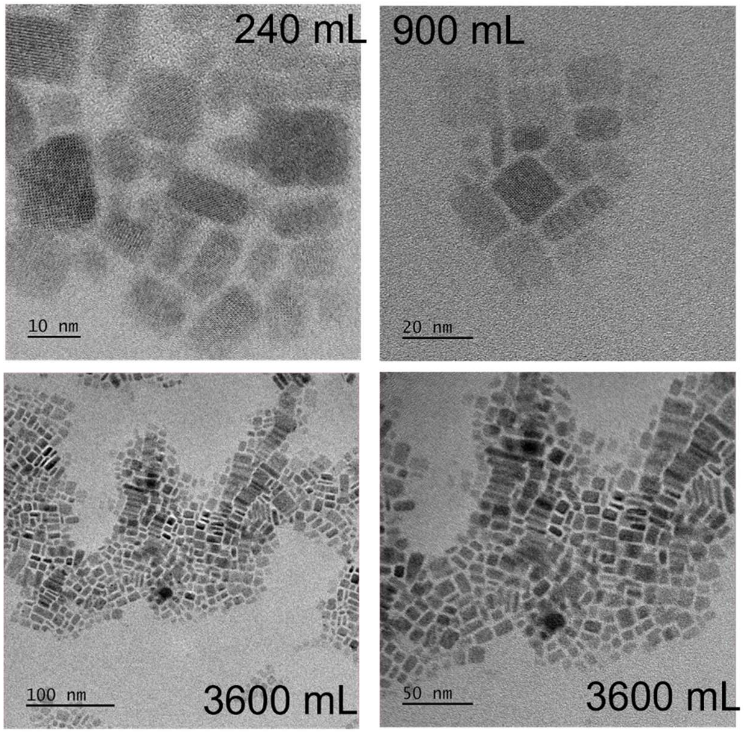

Figure S10. TEM images of NCs batches obtained at different volume scales (respectively, 900 mL on the top right, 240 mL on the top left and 3600 mL on the bottom) at different magnifications (reference bars are 10 and 20 nm respectively on the top and large field – 100 and 50 nm – on the bottom).

S12 - LINEAR MODEL FOR AMOUNT RECOVERED IN FUNCTION OF SYNTHESIS VOLUME

Table S5. The slope, the intercept and R^2 of the linear regression function used to represent the linear dependence of the weighted solid amount recovered of NCS in function of the volume of solution employed in synthesis.

Slope	Intercept	R^2
2.20 ± 0.03	6.4E-12 ± 2E-13	0.999

S13 – SYNTHESIS AND STEADY STATE OPTICAL CHARACTERIZATION OF $CsPbCl_3$ AND $CsPbBr_xCl_{3-x}$ NANOCRYSTALS

Synthetic procedure for $CsPbCl_3$ NCs.

Lead chloride ($PbCl_2$, 99.999%) was purchased from Merck. 60 mL of Solution A with cesium propionate in Heptane/iPrOH 2:1 v/v (3.3 mM) is prepared as reported in Methods for $CsPbBr_3$ NBs. For preparation of Solution B, $PbCl_2$ (1 mmol, 278 mg) is dissolved under magnetic stirring in a mixture of oleylamine (9 mmol, 2.67 mL), propionic acid (9 mmol, 0.67 mL) and isopropanol (0.67 mL) at 80 °C. After the complete dissolution of the precursors, the mixture is let cooling down to room temperature. Solution A is put under stirring with a Turbo-emulsifier homogenizer (15k RPM) in a 125 mL PP bottle, then Solution B is swiftly added. The mixture is let evolve for 30 s under homogenization. Stirring is stopped, and 30 mL of isopropanol are added to the crude solution to precipitate the nanocrystals. In this case, the precipitation doesn't happen immediately and is completed after 24 hours because the $CsPbCl_3$ formation is slower. After this time, obtained material is collected by centrifugation at 4500 RPM for 2 min. The supernatant is gathered for following recycle and the precipitate is dried in a dryer, weighted (70 mg) and finally stored in solid state in glove box under argon atmosphere**.**

Synthetic procedure for $CsPbBr_xCl_{3-x}$ NCs.

Br/Cl 1:1. 60 mL of Solution A with cesium propionate in Heptane/iPrOH 2:1 v/v (3.3 mM) is prepared as reported in Methods for $CsPbBr_3$ NBs. For preparation of Solution B, $PbCl_2$ (1 mmol, 278 mg) and $PbBr_2$ (1 mmol, 367 mg) are dissolved under magnetic stirring in a mixture of oleylamine (9 mmol, 2.67 mL), propionic acid (9 mmol, 0.67 mL) and isopropanol (0.67 mL) at 80 °C. The procedure is identical to the previous reported. In this case, iPrOH addition is followed by initial limited precipitation that does not happen in $CsPbCl_3$ sample, but it is completed in 24 hours as well. The supernatant is gathered for following recycle and the precipitate is dried in a dryer, weighted (80 mg) and finally stored in solid state in glove box under argon atmosphere**.**

Br/Cl 1:2. The procedure is identical to the previous one with the only difference in the use in Solution B of TBAB (1 mmol, 322 mg) instead of $PbBr_2$. In this way, we aim at tuning the halogen stoichiometry without adding further lead amount. Also in this case, the precipitation is limitedly triggered by iPrOH addition and ends after 24 hours. We obtained 85 mg of dried product.

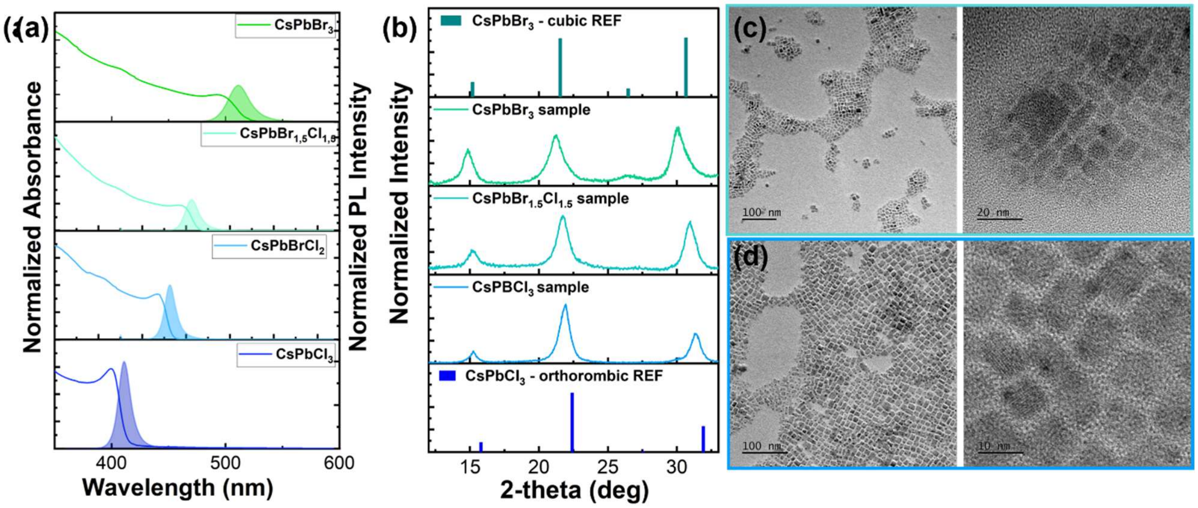

Figure S11. Spectroscopic characterization: (a) normalized absorption and photoluminescence spectra of $CsPbCl_3$, $CsPbBrCl_2$, $CsPbBr_{1.5}Cl_{1.5}$ and $CsPbBr_3$ respectively from the bottom to the top; Morphological characterization: (b) PXRD with references ($CsPbCl_3$ orthorombic -PDF Card No.: 01-075-0411 and $CsPbBr_3$ cubic - PDF Card No.: 01-084-0464) and (c) TEM images at different magnification of $CsPbBr_{1.5}Cl_{1.5}$ (greenwater line) and (d) $CsPbCl_3$ (light blue line).

S14 –$CsPbBr_3$ AND $CsPbCl_3$ WITH BUTYLAMINE (BuAM) LIGAND

Synthetic procedure for $CsPbBr_3$

Solution A of cesium propionate in Hept/iPrOH 2:1 is prepared as reported before. For Solution B, $PbBr_2$ (2 mmol, 731 mg) and TBAB (2 mmol, 639 mg) are dissolved at 80 °C in a mixture of BuAm (18 mmol, 1.77 mL) and PA (18 mmol, 1.34 mL) with 1.34 mL of iPrOH. The following procedure is the same as already described, except for the fact that NCs reprecipitate immediately without the addition of iPrOH. We recovered 310 mg of dried intense yellow solid: the amount is increased respect to the corresponding with OAm as ligand probably because lower colloidal stability induces more efficient precipitation of all the produced species. This aspect thus limits the proper in solution characterization.

Synthetic procedure for $CsPbCl_3$

Solution A of cesium propionate in Hept/iPrOH 2:1 is prepared as reported before. For Solution B, $PbCl_2$ (1 mmol, 278 mg) is dissolved at 80 °C in a mixture of BuAm (9 mmol, 0.88 mL) and PA (9 mmol, 0.67 mL) with 0.67 mL of iPrOH. The following procedure is the same as already described, except for the fact that NCs reprecipitate immediately with iPrOH addition. We recovered 110 mg of dried grey solid. Even in this case the recovered amount is higher with respect to the corresponding OAm ligand procedure.

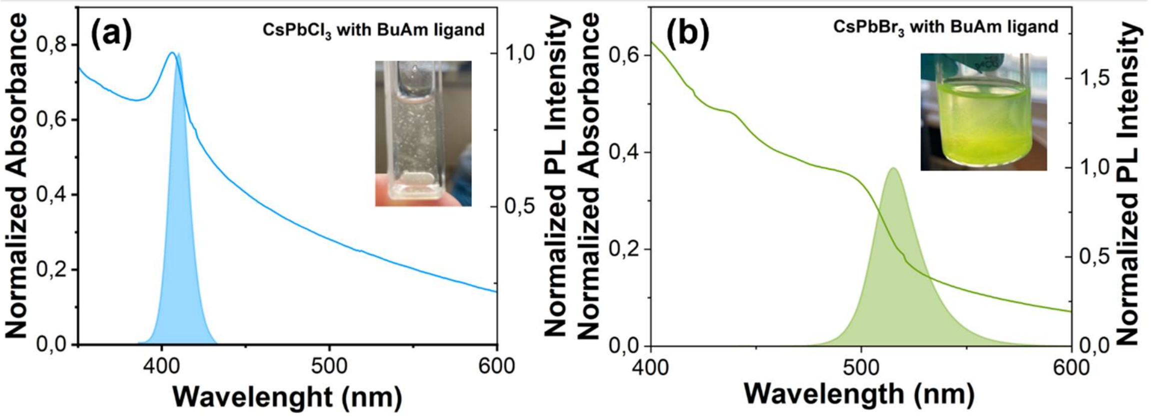

Figure S12. Normalized absorbance (only curve) and photoluminescence (filled area curve) and photograph of the resulting dispersion in toluene (1 mg/mL)(inset) of (a) $CsPbCl_3$ and (b) $CsPbBr_3$ samples synthetized with butylamine and propionic acid ligands.

S15 – RECOVERY: SUPERNATANT CHARACTERIZATION

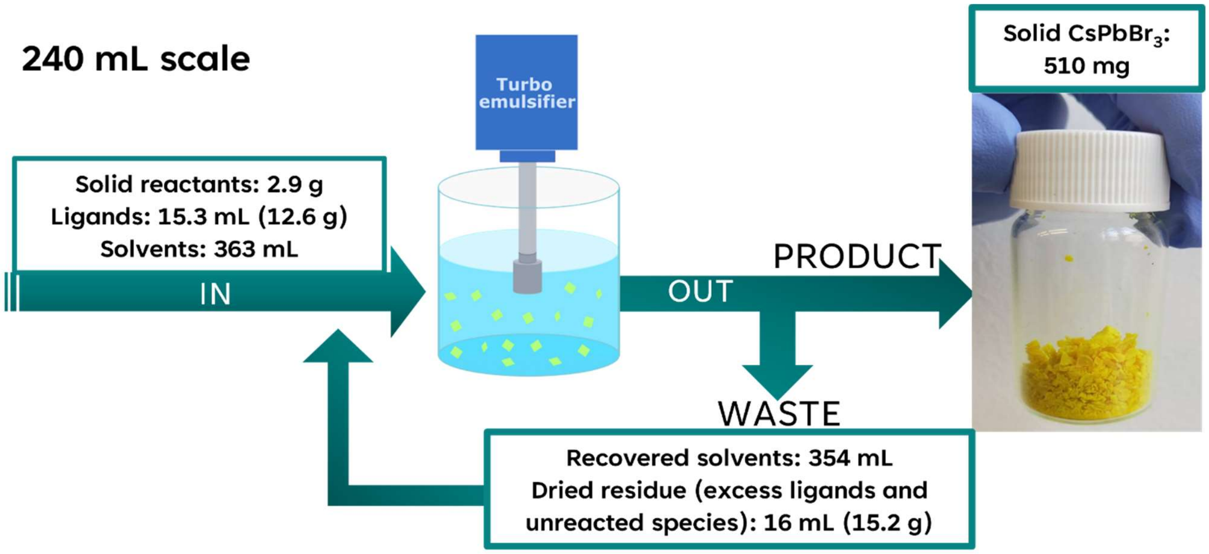

Scheme S1. Specific quantities involved in a 240 mL scale.

Table S6. Total amount of employed and recovered different species.

Species	Employed amount	Total employed amount	Recovered amount
**Solid reactants**	2.9 g	15.5 g	15.7 g
**Ligands**	12.6 g		
**Solvents**	363 mL	363 mL	354 mL

Supernatant treatment. After performing the reaction and centrifuging, the solid output is stored and the supernatant is distilled under reduced pressure, collecting 245 mL of a first fraction that distillates at 75 °C and 20 mL of a second at 83 °C. Given that Hept and iPrOH, the only two solvents employed, have an azeotrope concentration of 46:56 v/v with boiling point at 76.4 °C (1 atm) and the two solvents have respectively T_{bp} = 82.5 °C and 98.4 °C, we recovered 265 mL of Hept/iPrOH 1:1.3 v/v. The dried viscous residue is homogeneous and blue fluorescent and a white solid precipitates overnight. It can be dispersed again with heating under magnetic stirring at 80 °C: this solid is probably composed by $[PbBr_6]^{4-}$ aggregates and $CsPbBr_3$ clusters not recovered before for their dimensions under centrifugation limit. This dried residue composition was characterized by ^1H NMR in solution (C_6D_6, Figures S13), X-rays fluorescence (XRF, Figure S14(a)), TGA (7 wt% of residue at 490 °C, Figure S14(b)) and ICP-OES (Table S7).

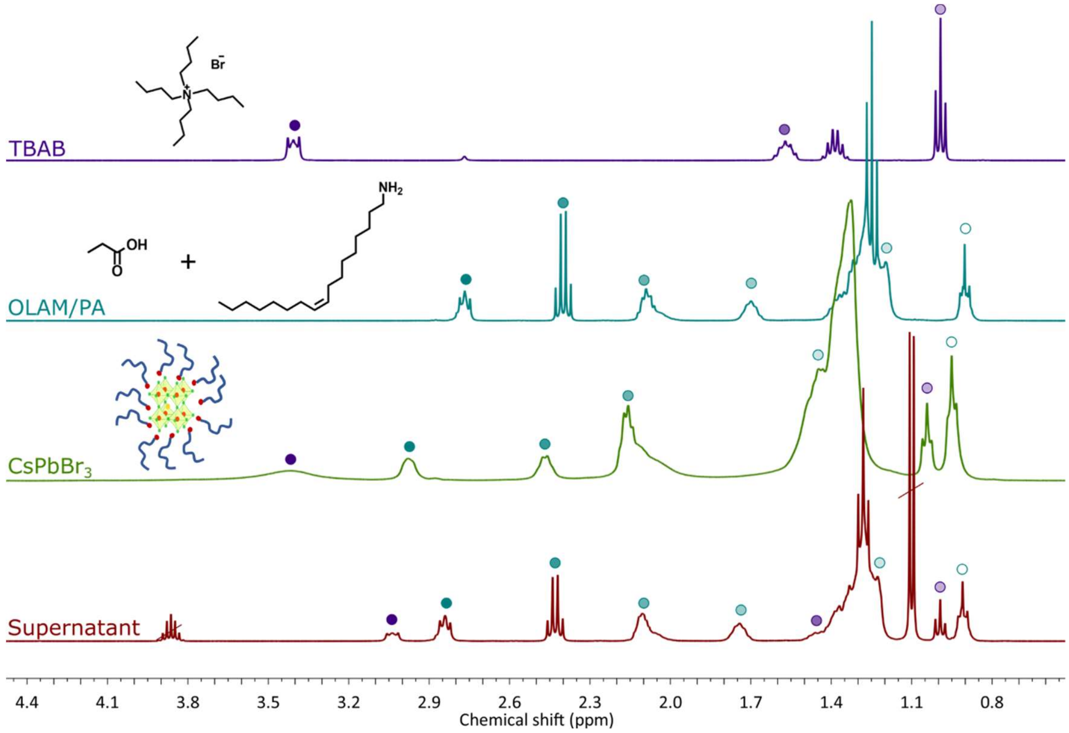

Figure S13. ^{1}H NMR in C_6D_6 of respectively: tetrabutylammonium bromide (TBAB), oleylamine (O*A*m)/propionic acid (PA) 1:1 mol ratio mixture, $CsPbBr_3$ sample and dried supernatant from synthesis.

In solution proton Nuclear Magnetic Resonance (NMR). ^{1}H NMR measurements were conducted at 300 K on a Bruker Avance NEO 400 MHz spectrometer equipped with BBFO probe. TBAB is directly dissolved in deuterated benzene (C_6D_6). OAm/PA 1:1 mol ratio are previously mixed under magnetic stirring and then dissolved in C_6D_6. $CsPbBr_3$ samples were dispersed from solid state directly in C_6D_6. The discarded supernatant was dried from iPrOH and Hept under reduced pressure and then a portion has been dissolved directly in C_6D_6. All samples were analysed at a concentration of 20 mg/mL in glass NMR tubes. All ^{1}HNMR spectra are referred to the signal of residual nondeuterated solvent (calibrating at 7.16 ppm singlet peak from benzene).

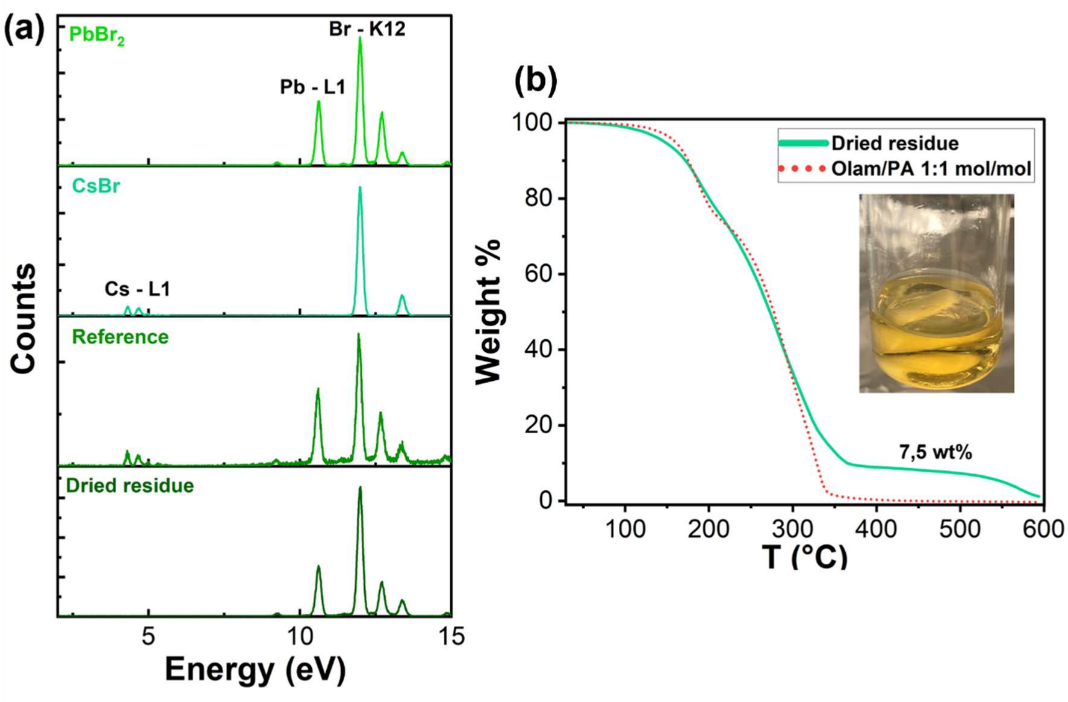

Figure S14. (a) X-ray fluorescence (XRF) spectra of dried residue, reference sample composed by weighted Cs_2CO_3 and $PbBr_2$ in OAm/PA 1:1 mol/mol, CsBr and $PbBr_2$. (b) TGA of the dried residue respect to ligands mixture and the photograph in the inset.

Table S7. From ICP-OES analysis of dried residue, sample weight and weight % of both cesium and lead are reported.

Sample	Weight	Cs wt%	Pb wt%
**Dried residue**	0.480 g	0.04	2.25

S16 –SYNTHESIS WITH RECOVERED REACTANTS

Table S8. Recovered and fresh amount added of the total needed for every species.

Species	Total amount needed	Recovered amount	Fresh amount
**Pb**	414 mg (2 mmol)	142 mg (0.68 mmol)	269 mg (1.3 mmol)
**Cs**	53.2 mg (0.4 mmol)	2.5 mg (0.02 mmol)	50.7 mg (0.38 mmol)
**Hept**	80 mL	30.3 mL	50 mL
**iPrOH**	40 + 1.3 + 60 mL	39.5 mL	0.9 + 60 mL
**OAm**	5.9 mL (18.4 mmol)	5 mL (15.8 mmol)	1 mL (3 mmol)
**PA**	1.3 mL (18.4 mmol)	1.6 mL (21 mmol)	0.1 mL (1.3 mmol)
**TBAB**	644 mg	441 mg	205 mg

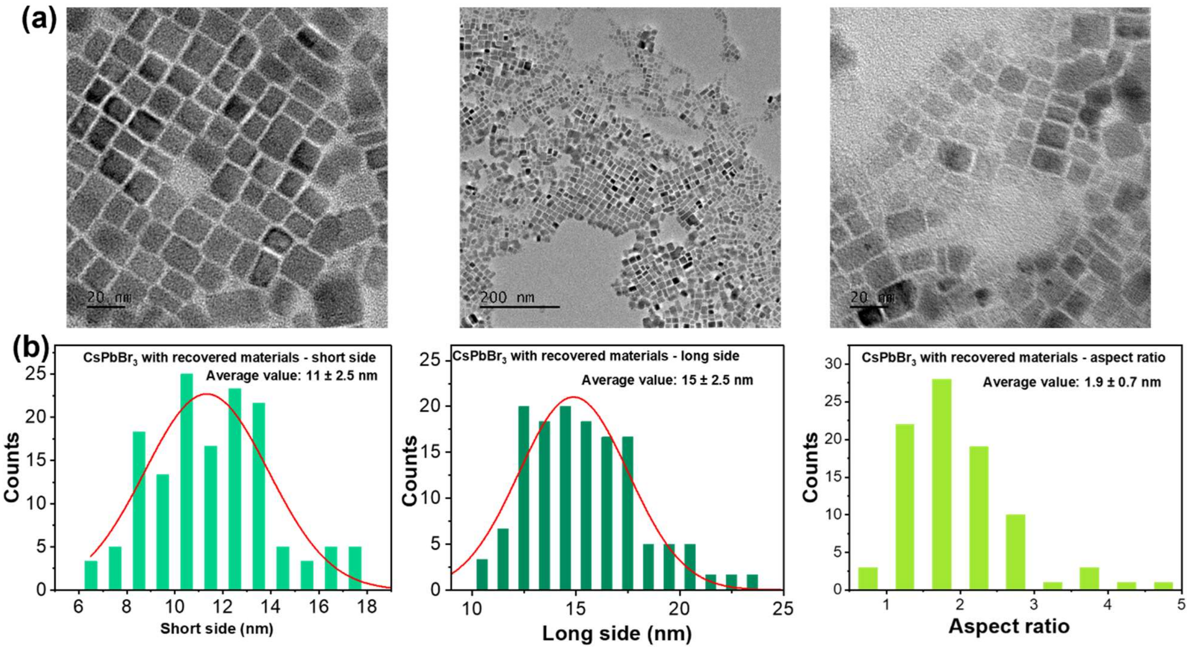

Figure S15. (a) TEM images that show different portions of the same sample and (b) their statistical analysis of long side, short side with gaussian fitting and aspect ratio of the NCs obtained with recovered reactants (on 106 NPs).

ACRONYMS

^{1}H NMR in solution proton nuclear magnetic resonance

ABS absoprtion

BuAm butylamine

DMF N,N'-dimethylformamide

DMSO dimethylsulfoxide

FWHM full-width at half maximum

Hept heptane

HRTEM high resolution transmission electron microscopy

ICP-OES inductively-coupled plasma optical emission spectroscopy

iPrOH isopropanol

LARP Ligand-assisted reprecipitation process

LHP-NC Lead halide nanocrystal

MSS magnetic stirring sample

NB nanobrick

NP nanoparticle

OAm oleylamine

PA propionic acid

PL photoluminesce

PXRD powder x-rays diffraction

QY quantum yield

RPM rotations per minute

TBAB tetrabutylammonium bromide

TES turbo-emulsified sample

REFERENCES

(1) Di Liberto, G.; Fatale, O.; Pacchioni, G. Role of Surface Termination and Quantum Size in α-CsPbX3 (X = Cl, Br, I) 2D Nanostructures for Solar Light Harvesting. Phys. Chem. Chem. Phys. 2021, 23 (4), 3031–3040. https://doi.org/10.1039/D0CP06245F.

(2) Yan, W.; Mao, L.; Zhao, P.; Mertens, A.; Dottermusch, S.; Hu, H.; Jin, Z.; Richards, B. S. Determination of Complex Optical Constants and Photovoltaic Device Design of All-Inorganic CsPbBr3 Perovskite Thin Films. Opt. Express 2020, 28 (10), 15706–15717. https://doi.org/10.1364/OE.392246.

(3) De Roo, J.; Ibáñez, M.; Geiregat, P.; Nedelcu, G.; Walravens, W.; Maes, J.; Martins, J. C.; Van Driessche, I.; Kovalenko, M. V.; Hens, Z. Highly Dynamic Ligand Binding and Light Absorption Coefficient of Cesium Lead Bromide Perovskite Nanocrystals. ACS Nano 2016, 10 (2), 2071–2081. https://doi.org/10.1021/acsnano.5b06295.
